# A very preterm infant born to mother of mirror syndrome secondary to fetomaternal hemorrhage: a case report

**DOI:** 10.1186/s12884-021-04179-5

**Published:** 2021-10-18

**Authors:** Sijie Song, Yefang Zhu, Gerhard Jorch, Xiaoting Zhang, Yan Wu, Wen Chen, Hua Gong, Ligang Zhou, Xueyan Wang, Xiaoyun Zhong

**Affiliations:** 1 Department of Neonatology, Chongqing Health Center for Women and Children, Longshan Road 120, Chongqing, China; 2 Department of Pediatrics, University Hospital Magdeburg, Magdeburg, Germany; 3 Department of Obstetrics, Chongqing Health Center for Women and Children, Chongqing, China

**Keywords:** Mirror syndrome, Fetomaternal hemorrhage, Etiology, Very preterm infant, Outcome

## Abstract

**Background:**

Mirror syndrome (MS) is defined as maternal edema with fetal hydrops and placental edema with different etiologies, such as rhesus isoimmunization and twin-twin transfusion syndrome. Herein, we showcased a unique MS case secondary to fetomaternal hemorrhage (FMH).

**Case presentation:**

A 32-year-old gravida 2 para 0 woman diagnosed with fetal hydrops was admitted to our hospital. Maternal laboratory tests revealed anemia, slightly increased creatinine and uric acid levels, hypoproteinemia, and significantly increased alpha-fetoprotein and hemoglobin-F levels. Therefore, FMH was diagnosed initially. Two days after admission, the woman had unexpectedly progressive anasarca and started to feel chest distress, palpitations, lethargy, and oliguria, and MS was suspected. An emergency cesarean section was performed to terminate the pregnancy. The maternal clinical symptoms and laboratory tests rapidly improved after delivery. A very preterm infant with a 2080-g birthweight at 31 weeks gestation survived after emergency cesarean section, active resuscitation, emergency blood transfusion, abdominocentesis, and advanced life support.

**Conclusions:**

FMH could develop into MS, providing new insights into the etiology of MS. Once MS is diagnosed, emergency cesarean section might be an alternative treatment. The very preterm infant survived with a favorable long-term outcome, and a well-trained perinatal work team is needed for such cases.

**Supplementary Information:**

The online version contains supplementary material available at 10.1186/s12884-021-04179-5.

## Background

Mirror syndrome (MS), also called Ballantyne syndrome, was first described as maternal edema associated with fetal hydrops and placental edema due to rhesus (Rh) isoimmunization by Ballantyne in 1982 [1]. MS is a rare syndrome with different etiologies (Rh isoimmunization, TTTs, and viral infection), and the mortality of the fetus is extremely high [2, 3]. Interestingly, the leading cause of MS in China is thalassemia (Bart’s hydrops fetalis syndrome), which differs from previous reports worldwide [4, 5]. The incidence rate of MS in China is approximately 0.014–0.047%, and few fetuses have survived until now [4, 5].

Fetomaternal hemorrhage (FMH) is defined as a loss of fetal blood cells to the maternal circulation. FMH can lead to severe fetal anemia and/or fetal hydrops [6]. However, to date, no study has reported that MS is related to FMH [2–5]. Herein, for the first time, we reported a unique MS case secondary to FMH, which would provide new insights into the etiology of MS. Although the infant suffered from a long course of severe edema and anemia during the pregnancy, the very preterm infant (≦ 32 weeks of gestation) with a 2080-g birthweight survived with a good long-term outcome under the coordination of our perinatal center.

## Case presentation

### Maternal history

A 32-year-old woman, gravida 2 para 0 (two induced abortions), in vitro *fertilization*, group A, rhesus-positive was referred at 31^+ 2^ weeks gestation to our hospital due to fetal hydrops for 10 days. She had hypothyroidism during pregnancy (treated with levothyroxine). The screening test for Down’s syndrome showed low risk, and the ultrasound proved the fetus had no malformation in the second trimester. Physical examination showed 70 kg body weight and apparent edema in both legs with a peripheral blood pressure of 133/63 mmHg.

The ultrasound showed severe ascites (the deepest depth = 6.77 cm), mild edema and subcutaneous edema of the fetus (depth 0.59 cm), thickened placenta (5.8 cm), polyhydramnios (amniotic fluid index = 24.75 cm, reference value 5–18 cm), and increased peak systolic velocity of the middle cerebral artery (> 2.0 MoM, reference value < 1.5 MoM). Comprehensively, fetal anemia was considered. Maternal laboratory tests revealed anemia (100 g/L), slightly increased creatinine (91 μmol/L) and uric acid (559 μmol/L) levels, hypoproteinemia (albumin 19 g/L), and significantly increased alpha-fetoprotein (AFP, 4864.5–5005.4 ng/ml) and hemoglobin-F (HbF, 4.3%) levels. Other tests were all normal (cardiac markers, coagulation function, electrolytes) or negative (thalassemia genetic test, TORCH, HIV, syphilis, hepatitis B and C). FMH was diagnosed initially. Given that the fetus was at less than 34 weeks of gestation, dexamethasone and magnesium sulfate were used to promote lung maturation and brain protection [7].

Unexpectedly, the maternal body weight increased by 6 kg (76 kg) within 2 days after admission, and she showed anasarca and started to feel chest distress, palpitation, lethargy, and oliguria (200 ml in 15 h). Preeclampsia was excluded because routine blood tests showed hemodilution. The ultrasound still showed severe ascites, edema and subcutaneous edema of the fetus, thickened placenta (5.2 cm), polyhydramnios (amniotic fluid index = 26.5 cm), increased peak systolic velocity of the middle cerebral artery (> 2.0 MoM), tricuspid regurgitation (mild), and increased cardiothoracic ratio (0.44, reference value 0.25–0.35). The fetal heart rate was normal before termination of the pregnancy. MS was diagnosed on the basis of progressive maternal edema secondary to fetal hydrops and placental edema. MS is reported to have high mortality of the fetus [2, 3], so emergency cesarean section was considered after a discussion involving the ICU, ultrasound team, and the neonatology, obstetrics, and anesthesiology departments. In addition, an experienced resuscitation team consisting of neonatologists, nurses and midwives was alerted for resuscitation and subsequent treatment.

The maternal clinical symptoms rapidly improved postnatally, and edema completely disappeared 4 days after cesarean section. After delivery, albumin was administered for 2 days for amelioration of hypoproteinemia, and furosemide and heparin were used for oliguria and hypercoagulability, respectively, for 5 days. Maternal AFP levels were reduced to 1278.2 ng/ml 6 days after delivery. The woman was discharged 6 days after the surgery.

### Infant’s medical history

A girl with a 2080-g birthweight was delivered at 31^+ 4^ weeks gestation. Amniotic fluid 2500 ml, clear; grossly edematous placenta (890 g, 18 × 18 × 5 cm^3^) (Additional file 1); other labor history was normal.

#### Resuscitation

After birth, the baby showed no breath, hypomyotonia and paleness. Positive pressure ventilation (PPV), endotracheal intubation, chest compressions and epinephrine infusion were performed. Notably, a bolus of 20 ml saline (within 10–15 min) was infused at 30 s after birth because hypovolemia was suspected due to severe anemia. The resuscitation process is shown in Additional file 2. Apgar scores were 2, 5, and 5 points at 1, 5 and 10 min, respectively. Then, the baby was transferred to the NICU under PPV via endotracheal intubation (FiO_2_ 100%).

#### Treatment and laboratory tests

**Fig. 1:**
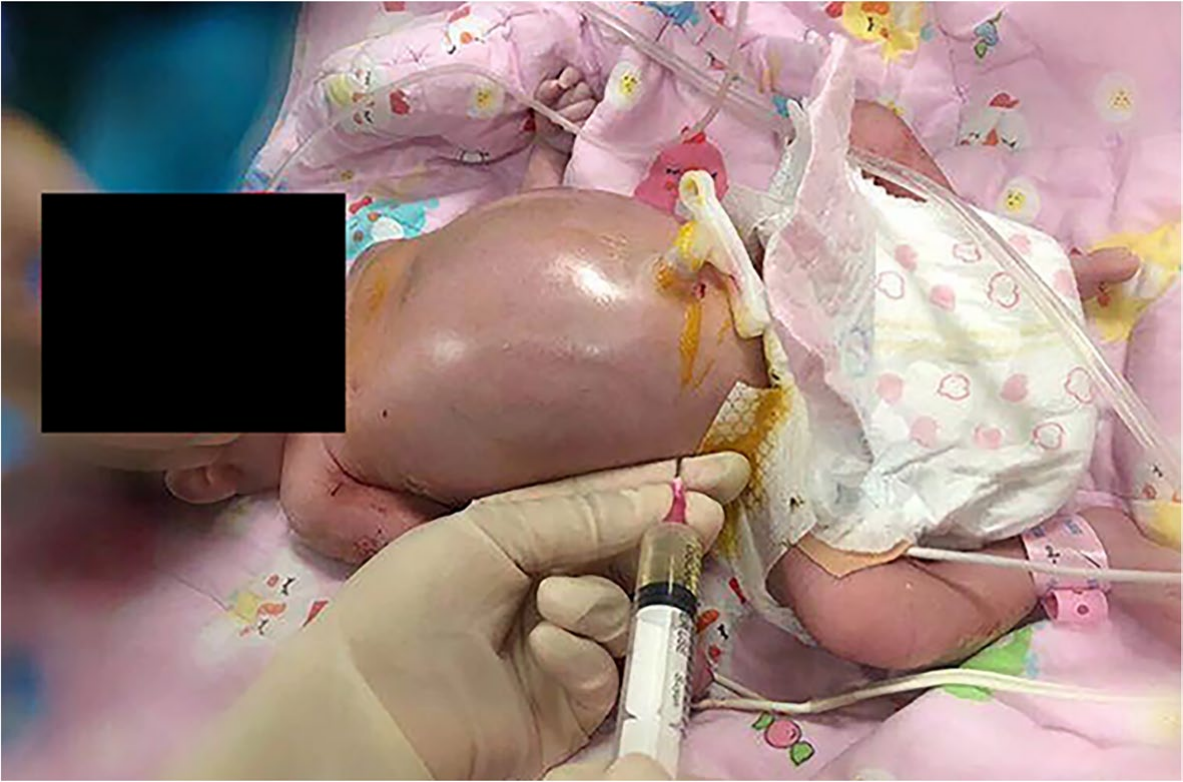
The edema/ascites and rescue process of the infant in the NICU

Spontaneous breathing of the baby appeared 17 mins after birth. Upon admission, the baby had a respiratory rate of 50–60 times/min, heart rate 144 beats/min, and peripheral blood pressure 75/48 (58) mmHg. Emergency ultrasound showed severe seroperitoneum with a maximum depth of 2.7 cm, mild hydrothorax, and no malformation of abdominal organs. To relieve respiratory distress, abdominocentesis was performed 19 min after birth, and 122 ml of light-yellow ascites was drawn out (Fig. 1). The laboratory tests of ascites indicated exudates (with 85% lymphocytes).

Blood analysis and blood gas showed severe anemia, metabolic acidosis and hyperlactatemia (hemoglobin (Hb): 58 g/L, pH: 7.045, base excess: − 14.1 mmol/L, and lactate: 9.7 g/L) (Additional file 3). Therefore, a rapid fluid replacement of 20 ml saline (10 mL/kg) was infused in 30 min, and a 70-ml red blood cell suspension (Type O, RhD positive) was transfused 48 mins after birth. Conventional ventilation (assist/control mode) was provided, and the initial settings were FiO_2_ 100%, MAP 10–11 cmH_2_O, PIP/PEEP 20/8 cmH_2_O, RR 45, tidal volume 9–10 ml, and minute volume 0.4–0.5 L/min. Soon thereafter, the blood gas returned to normal gradually, the symptoms of anemia resolved, and Hb levels recovered. Approximately 4 h after birth, the ventilator settings were adjusted to FiO_2_ 28–25%, MAP 8–9 cmH_2_O, PIP/PEEP 18/7 cmH_2_O, RR 40, tidal volume 8–9 ml, minute volume 0.3–0.4 L/min. More than 19 h after birth, the baby was extubated and supported by a humidified high-flow nasal cannula (HHFNC, FiO_2_ 40–21%, flow 4–2 L/min) for 5 days.

To demonstrate the diagnosis of FMH, a series of etiological tests were performed in the infant (Table 1). Cardiac ultrasound showed patent ductus arteriosus (2.6 mm), patent foramen ovale, tricuspid regurgitation (mild), mitral regurgitation (mild), pulmonary hypertension (46 mmHg), and the ejection fraction was normal. There was also no clinical sign of heart failure. And abdominal ultrasound proved no malformation or teratoma. All the main causes of MS or fetal hydrops were excluded [2–5].

**Table 1:** Anemia and edema etiology tests of the infant

Item	Results
Thalassemia gene screening	negative
G-6-PD enzyme activity	2.01 (reference value: 1.00–2.30)
Parvovirus B19	negative
TORCH	CMV-IgG: positive, others: negative
Chromosome analysis	normal (46, XY)
hemolytic test	not accurate (performed after blood transfusion)

1. *G-6-PD* Glucose-6-phosphate dehydrogenase, *TORCH* toxoplasmosis, others, rubella, cytomegalovirus and herpes simplex virus. 2. All these tests were performed postpartum

#### Nervous system monitoring

We paid close attention to the neurological symptoms with regard to long-lasting severe anemia and hypoxemia both in utero and after delivery. The baby showed no clinically abnormal neurological symptoms during hospitalization. Cranial ultrasound on days 1, 2, 4 and 11 after birth revealed an increased resistance index (RI) of the middle cerebral artery but no hypoxic or ischemic changes (edema, hemorrhage, etc.) and no signs of periventricular leukomalacia (PVL). Moreover, the amplitude integrated electroencephalogram (aEEG) on the second day after birth showed that a sleep cycle appeared, a discontinuous quiet sleep period, and no seizures under video monitoring, which were all normal for a preterm infant at 31^+ 5^ weeks gestation. Assessment of general movements (GMs) (performed 13 days after birth) showed normal movement of the body. Both ears passed the automatic auditory brainstem response (AABR) test in the hearing screen. At 34^+ 5^ weeks of corrected gestation before discharge, brain magnetic resonance imaging (MRI) presented a normal structure according to gestation, and no signs of PVL or other abnormalities were noted. After 24 days of hospitalization (34^+ 6^ gestation), the baby was discharged with a body weight of 1810 g, body length of 40.4 cm, and head circumference of 28.5 cm. All of these values were less than the 10th percentiles, implying extrauterine growth restriction (EUGR).

#### Long-term follow up

After discharge, we conducted a long-term clinical follow-up for this very preterm infant. Physical development was assessed using the Fenton preterm growth chart for girls [8]. Although the infant had EUGR at discharge, the body weight and length gradually increased close to the 50th percentiles, and the head circumference was slower to catch-up but around the 15th percentiles at 1–5 months postmenstrual age (PMA). Regarding neurological assessments, we performed the Alberta Infant Motor Scale (AIMS) [9] at 4 months PMA and the Bayley Scales of Infant Development-I (BSID-I) [10] at 5 and 15 months PMA. The results showed 10th–25th percentiles for AIMS. BSID-I scores were 82 for Mental Development Index (MDI) and 83 for Psychomotor Development Index (PDI) at 5 months PMA followed by 91 for MDI and 89 for PDI at 15 months PMA. Above all, this baby only had a suspicious delay in neurodevelopmental evaluation at 5 months PMA that returned to normal at 15 months PMA.

## Discussion and conclusions

MS is defined as progressively maternal edema associated with fetal hydrops and placental edema with different etiologies. Reviews have shown that the leading causes of MS involve rhesus isoimmunization, TTTs, viral infection, fetal malformations or tumors [2, 3]. According to Chinese literature (Additional file 4), the leading cause of MS in China is thalassemia (Bart’s hydrops fetalis syndrome), which differs from that noted in previous studies. No study has reported that MS could occur secondary to FMH. Therefore, to the best of our knowledge, our case study is the first to report MS secondary to FMH, providing new insights into the etiology of MS. In our case, the clinical symptoms, laboratory tests, and rapid improvement of the mother after delivery supported the diagnosis of MS. Simultaneously, maternal laboratory examination (AFP and HbF significantly increased before labor but rapidly decreased after delivery) also supported the diagnosis of FMH.

The mortality of the fetus is extremely high in this condition. In developed countries, the overall rate of intrauterine death is 56–67.26% [2, 3]. Unfortunately, the mortality in China is much higher [4, 5]. One of the major reasons for the high mortality in China is that the main leading cause of MS is thalassemia (Bart’s hydrops fetalis syndrome), for which there is no effective intrauterine treatment. The causes of survival were all TTTs: 1 patient delivered at 36 weeks gestation, and the other 4 patients underwent fetoscopic surgery between 33 and 36 weeks (Additional file 4). Our case is the first very preterm infant who survived MS in China.

MS is a reversible disease. Once the cause is treated and the fetal hydrops improves or the pregnancy is terminated, maternal symptoms disappear [11, 12]. In our case, FMH was the primary cause; therefore, intrauterine transfusion (IUT) might represent an appropriate option to alleviate fetal hydrops [6]. However, we do not have sufficient experience with IUTs. Fetuses with severe fetal anemia and hydrops might not tolerate IUTs; thus, the possibility of fetal death would increase [13–15]. Whether outcomes could be improved by IUT is unknown [13]. Therefore, emergency termination of pregnancy by cesarean section is preferred in this case. The mother recovered soon after surgery, demonstrating that termination of pregnancy is an effective treatment for MS.

One noteworthy phenomenon is that although we provided PPV and mechanical ventilation to the infant immediately after birth, the blood gas still presented distinct metabolic acidosis and hyperlactatemia, which were eliminated soon after blood transfusion and volume expansion. The main reason is that severe anemia and hypovolemia lead to poor oxygenation and perfusion. Blood gas improved soon after treatment, which is critical in rescue. The reticulocyte percentage after birth was significantly increased 4-fold (29.4%) the reference value (approximately 7% at birth) and gradually decreased after Hb was corrected, indicating chronic intrauterine anemia.

Kadooka et al. demonstrated that the pH was significantly lower and the base deficit was significantly greater in the poor outcome group of FMH, but the initial fetal Hb is the most valuable prognostic factor to predict further outcomes for FMH infants [16, 17]. Favorable long-term outcomes are expected in FMH infants with an initial Hb greater than 4.5 g/dL. An Hb level of 4.5 g/dL represents approximately 25% of the normal level, and an animal experiment showed that a decrease of greater than 50% in blood oxygen was associated with a decrease in blood pH and decreased oxygen in the brain [18]. However, the outcome of infants with FMH may also depend on whether the event is acute or chronic [19]. In our case, intrauterine chronic anemia was noted, and Hb levels were greater than 4.5 g/dL. In addition, the brain structure imaging results were normal, and neurological assessments at 15 months were almost normal. All of the above factors likely indicate a favorable prognosis; thus, a good outcome will most likely be achieved in the long term. Nevertheless, very preterm infants are associated with higher risks for motor, learning, and behavior problems [20, 21]. Therefore, we will continue the long-term follow-up. Poor infant outcomes, such as cerebral palsy, mental retardation, and epilepsy, will be closely monitored.

Some experiences encountered during the treatment of MS in a very preterm infant should be shared. First, perinatal teamwork and multidisciplinary cooperation for maternal and infant safety are significant. In this case, these steps, including prenatal consultation, promotion of lung and brain maturation by dexamethasone and magnesium sulfate and preparation of resuscitation, are essential and significant. Second, timely termination of the pregnancy and an active resuscitation strategy are also the most critical steps. Third, postnatal emergency treatment included blood transfusion, abdominocentesis, and advanced life support. Finally, a relatively mature gestational age provided us with objective conditions and hope for successful management.

In conclusion, the diagnosis of MS is tricky, and its pathogenesis and pathophysiology are unknown. Our case demonstrated that FMH could develop into MS, providing additional information on the etiology of MS and thus new diagnostic thoughts for clinicians in the future. Moreover, this case highlights the unexpected change during FHM, reminding us of the need to better understand the clinical manifestations of FHM. Both MS and severe FHM are dangerous for the mother and fetus. For maternal edema with fetal hydrops, we should actively look for evidence regarding whether MS secondary to FHM is the cause to formulate the appropriate therapeutic timing and strategy, which might be the key step to achieving the best prognosis for the mother and fetus. If there is no opportunity for intrauterine treatment, timely termination of the pregnancy and a well-trained perinatal work team are needed to achieve a favorable long-term outcome for the very preterm infant.

## Supplementary Information

**Additional file 1.** The grossly edematous placenta.

**Additional file 2.** The resuscitation process. Note: PPV: positive pressure ventilation, UVC: umbilical venous catheter.

**Additional file 3.** Blood analysis and blood gas of the infant.

**Additional file 4.** Chinese literature review with case reports of mirror syndrome.

## Data Availability

All data generated or analyzed during this study are included in this article.

## Abbreviations

MS: Mirror syndrome
FMH: Fetomaternal hemorrhage
AFP: Alpha-fetoprotein
Hb: Hemoglobin
PPV: Positive pressure ventilation
FiO2: Fraction of inspired oxygen
MAP: Mean airway pressure
PIP: Peak inspiratory pressure
PEEP: Positive expiratory pressure
RR: Respiratory rate
HHFNC: Humidified high flow nasal cannula
PVL: Periventricular leukomalacia
aEEG: Amplitude integrated electroencephalogram
GMs: General movements
AABR: Automatic auditory brainstem response
MRI: Magnetic resonance imaging
EUGR: Extrauterine growth restriction
AIMS: Alberta infant motor scale
BSID-I: Bayley scales of infant development-I
MDI: Mental development index
PDI: Psychomotor development Index
DQ: Developmental quotient
IUT: Intrauterine transfusion
TTTs: Twin-twin transfusion syndrome
MODS: Multiple organ dysfunction syndrome
DIC: Disseminated intravascular coagulation
UVC: Umbilical vein catheter
PMA: Postmenstrual age
NICU: Neonatal intensive care unit
TORCH: Toxoplasmosis, others, rubella, cytomegalovirus and herpes simplex virus
