# Supplementary figures and images for: A very preterm infant born to mother of mirror syndrome secondary to fetomaternal hemorrhage: a case report
Source: BMC Pregnancy Childbirth. 2021 Oct 18;21:701. doi: 10.1186/s12884-021-04179-5 (PMC8522257; doi:10.1186/s12884-021-04179-5)

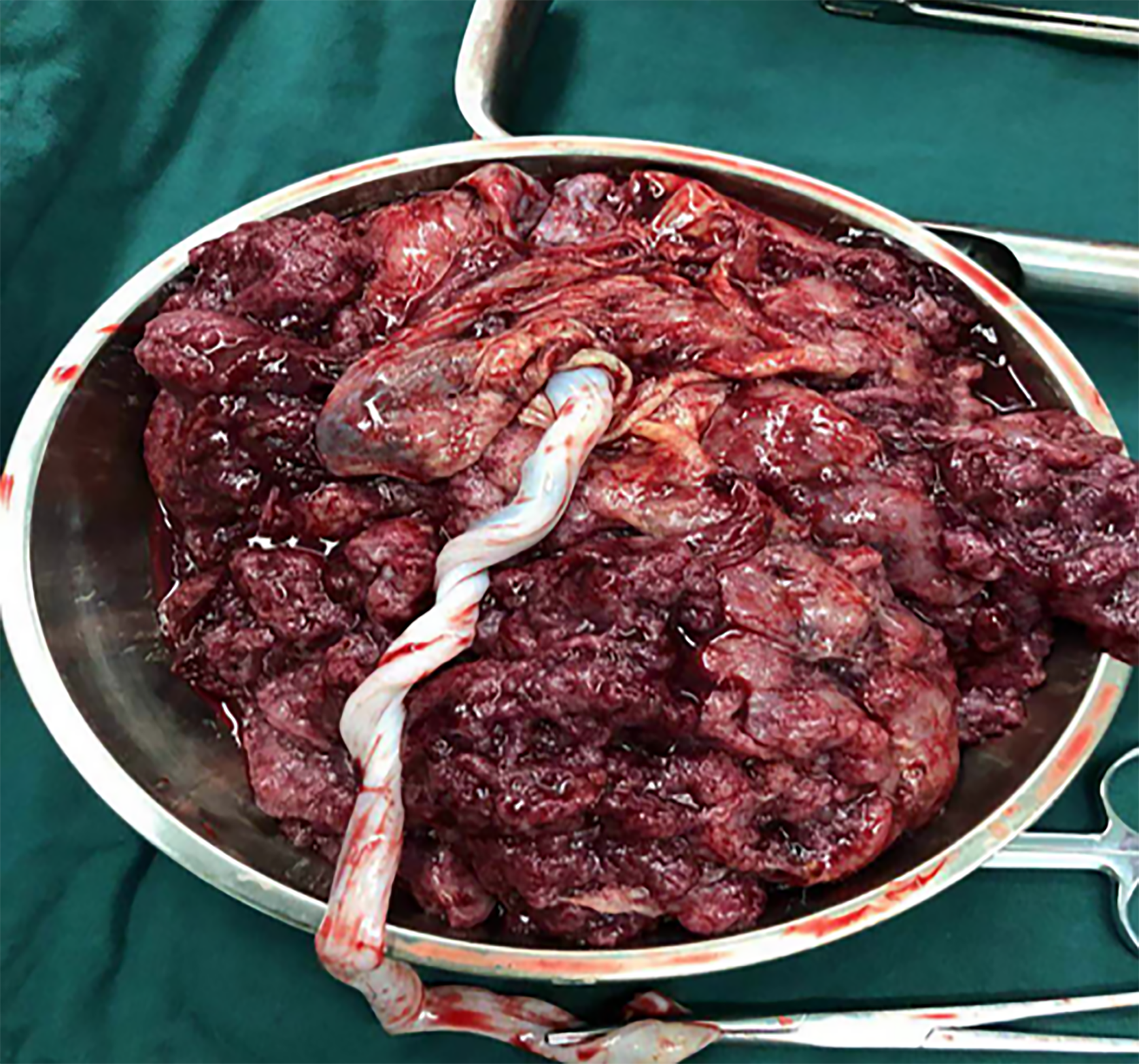

Supplement: Supplementary file 1 — Additional file 1. The grossly edematous placenta. [file 12884_2021_4179_MOESM1_ESM.png]

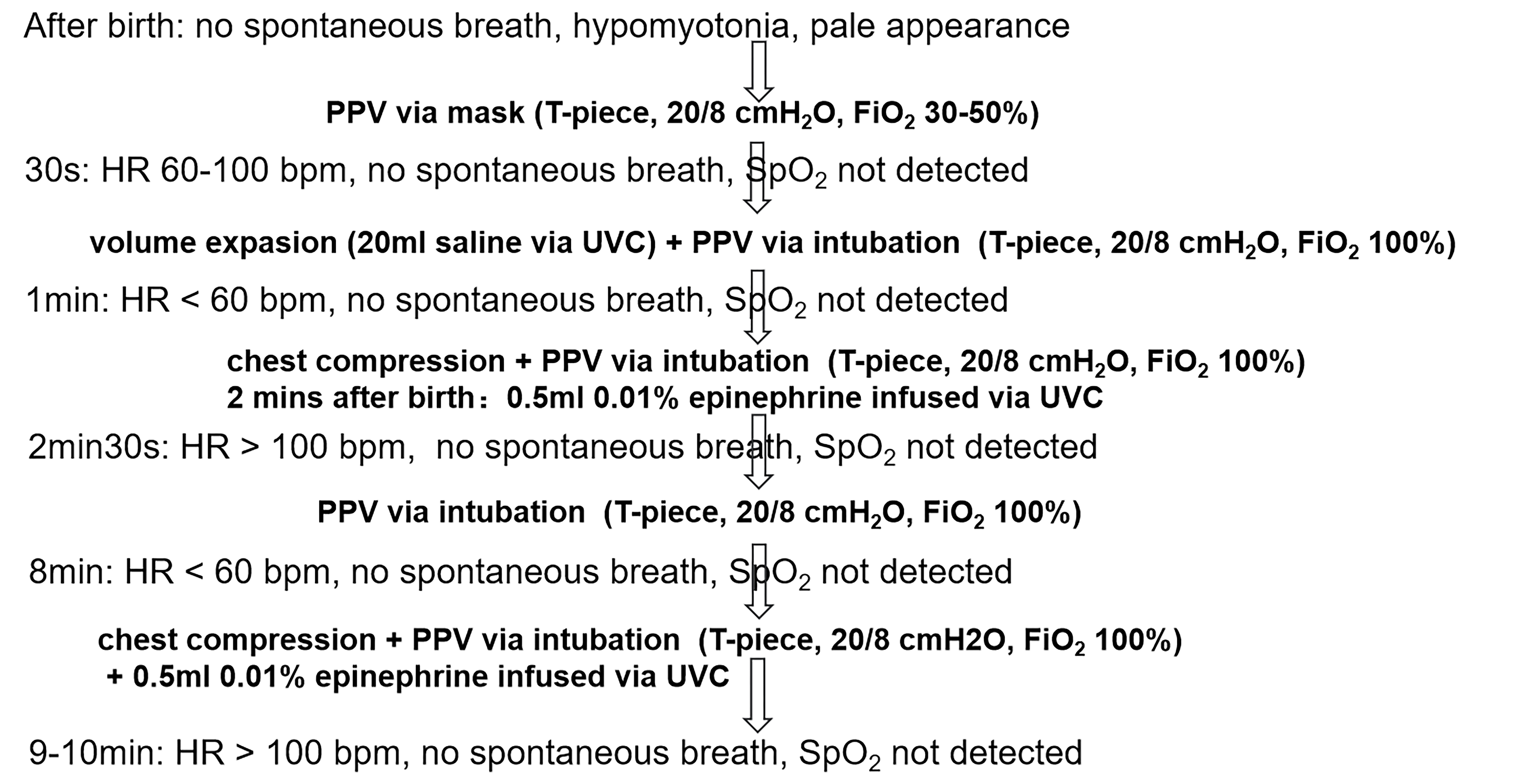

Supplement: Supplementary file 2 — Additional file 2. The resuscitation process. Note: PPV: positive pressure ventilation, UVC: umbilical venous catheter. [file 12884_2021_4179_MOESM2_ESM.png]
