# Supplementary material for: A very preterm infant born to mother of mirror syndrome secondary to fetomaternal hemorrhage: a case report
Source: BMC Pregnancy Childbirth. 2021 Oct 18;21:701. doi: 10.1186/s12884-021-04179-5 (PMC8522257; doi:10.1186/s12884-021-04179-5)
Supplement: Supplementary file 3 — Additional file 3. Blood analysis and blood gas of the infant. [file 12884_2021_4179_MOESM3_ESM.doc]

**Table S1. Blood analysis and blood gas of the infant**

**Blood analysis**

| **Time** | **WBC**  **(10^9^/L)** | **PLT**  **(10^9^/L)** | **Hb**  **(g/L)** | **CRP**  **(mg/L)** | **PCT**  **(ng/mL)** | **I/T** | **Ret (%)** |
| --- | --- | --- | --- | --- | --- | --- | --- |
| **0h (Umbilical vein)** | 6.8 | 307 | **58** |  |  |  | **29.3%** |
| **1-d** | 11.1 | 307 | **58** | <1.67 |  | 18 | **29.43%** |
| **1+d** | 10.0 | 179 | 121 | 2.68 |  |  | 11.57% |
| **3+d** | 14.4 | 208 | 139 | 2.26 | 0.53 | 6 | 11.75% |
| **5+d** | 14.4 | 284 | 148 | <1.67 |  |  | 7.8% |
| **10+d** | 16.7 | 383 | 138 | <1.67 |  |  | 3.53% |

**Blood gas**

| **Time** | **FiO_2_** | **pH** | **pO_2_** | **pCO_2_** | **HCO_3_** | **BE** | **Glu** | **Lac** | **Hb** | **Hct** |
| --- | --- | --- | --- | --- | --- | --- | --- | --- | --- | --- |
| **0h (Umbilical vein)** |  | 7.280 | 23 | 48 | 22.6 | -4.1 | 4.9 | 3.2 | **36.0** | **15%** |
| **1h** | 30% | **7.045** | 45.3 | 55.2 | 15.1 | **-14.1** | 5.1 | **9.7** | **57** | **17.4%** |
| **2+h** | 21% | 7.31 | 90.7 | 29.1 | 14.6 | **-10.5** | 1.8 | **6.5** | **95** | **29.2%** |
| **19h** | 25% | 7.308 | 78.1 | 42.4 | 21.2 | -4.9 | 3.3 | 1.1 | 121 | 37.2% |
| **1d19h** | 21% | 7.364 | 97.1 | 34.6 | 19.7 | -5.7 | 4 | 1 | 150 | 46.1% |

Note: WBC: white blood cell, PLT: platelet, Hb: hemoglobin, CRP: C-reactive protein, PCT: procalcitonin, I/T: immature: total neutrophil proportion, Ret: reticulocyte
