# Supplementary material for: A very preterm infant born to mother of mirror syndrome secondary to fetomaternal hemorrhage: a case report
Source: BMC Pregnancy Childbirth. 2021 Oct 18;21:701. doi: 10.1186/s12884-021-04179-5 (PMC8522257; doi:10.1186/s12884-021-04179-5)
Supplement: Supplementary file 4 — Additional file 4. Chinese literature review with case reports of mirror syndrome. [file 12884_2021_4179_MOESM4_ESM.doc]

Table S2. Chinese literature review with case reports of Mirror Syndrome.

| Paper No. | Time of maternal symptoms or diagnosis (weeks) | Fetal pathology | Fetal symptoms | Maternal manifestations or complications | Gestation at delivery (weeks) | Fetal outcome |
| --- | --- | --- | --- | --- | --- | --- |
| 1 | 21 - 33 | n = 8  7: thalassemia (Bart's hydrops fetalis syndrome)  1: TTTs | hydropic placenta or placenta megaly or thickened placenta , pericardial effusion(7/8), hydrops fetalis(7/8), ascites (4/8), hydrothorax(3/8), | **Manifestations:** edema, anemia and hemodilution, hypoproteinemia, hypertension (6/8), proteinuria (6/8), elevated creatinine (1/8)  **Complications:** [postpartum](../../../../D:/Dict/8.8.0.0/resultui/html/index.html#/javascript:;) [hemorr](../../../../D:/Dict/8.8.0.0/resultui/html/index.html#/javascript:;)- hage (5/8), heart failure(4/8), pulmonary edema (2/8) | 21 - 34 | intrauterine death |
| 2 | 16^+5^ - 34^+1^ | n = 20  12: thalassemia (Bart's hydrops fetalis syndrome)  8: unknown causes | hydropic placenta or placenta megaly or thickened placenta, hydrops fetalis, ascites , hydrothorax | **Manifestations:** edema, anemia and hemodilution, proteinuria, hypoproteinemia, elevated uric acid, hypertension (14/20), elevated liver enzymes (3/20)  **Complications:** heart failure and pulmonary edema (4/20), [postpartum](../../../../D:/Dict/8.8.0.0/resultui/html/index.html#/javascript:;) [hemorrhage](../../../../D:/Dict/8.8.0.0/resultui/html/index.html#/javascript:;) (7/20, 4 out of 7 > 1000 ml) | 26^+2^ - 36^+3^ | intrauterine death or stillbirth |
| 3 | not reported | n = 9  8: thalassemia (Bart's hydrops fetalis syndrome)  1: heart malformation | hydropic placenta, placenta megaly, thickened placenta, hydrops fetalis, ascites , hydrothorax, pericardial effusion, oligohydramnion (2/9) | **Manifestations:** edema, anemia and hemodilution, elevated uric acid, hypoproteinemia and proteinuria (8/9), hypertension (5/9)  **Complications:** placenta adherence and [postpartum](../../../../D:/Dict/8.8.0.0/resultui/html/index.html#/javascript:;) [hemorrhage](../../../../D:/Dict/8.8.0.0/resultui/html/index.html#/javascript:;) (4/9, 3 out of 4 > 1000 ml), MODS (1/9) | 28^+5^ - 33^+6^ | 7: intrauterine death  2: died several mins after delivery |
| 4 | 24^+3^ - 36^+2^ (diagnosis) | n = 21  15: thalassemia (Bart's hydrops fetalis syndrome)  2: heart malformation  1: TTTs  1: Pavovirus B19  2: unknown causes | thickened placenta, hydrops fetalis | **Manifestations:** edema, anemia and hemodilution (20/21), hypoproteinemia (19/21), elevated uric acid (19/21), proteinuria (18/21), hypertension (10/21), thrombocytopenia (3/21), headache (1/21)  **Complications:** [postpartum](../../../../D:/Dict/8.8.0.0/resultui/html/index.html#/javascript:;) [hemorrhage](../../../../D:/Dict/8.8.0.0/resultui/html/index.html#/javascript:;) (12/21, 4 out of 12 > 3500 ml), placenta adherence (8/21), pulmonary edema (6/21), placental abruption (3/21), DIC (1/21) | not reported | 16: intrauterine death  4: died after delivery  1 (TTTs): 36+2 weeks cesarean section, survived |
| 5 | 29.77 ± 1.6 (diagnosis) | n = 15  14: thalassemia (Bart's hydrops fetalis syndrome)  1: unknown causes | hydropic placenta, thickened placenta, hydrops fetalis, ascites, oligohydramnion (5/15) | **Manifestations:** edema, anemia and hemodilution, hypoproteinemia, elevated uric acid (13/15), oliguria or anuria (5/15), hypertension (3/15), headache (1/15), epigastric pain (1/15)  **Complications:** [postpartum](../../../../D:/Dict/8.8.0.0/resultui/html/index.html#/javascript:;) [hemorrhage](../../../../D:/Dict/8.8.0.0/resultui/html/index.html#/javascript:;), placenta adherence (6/15), acute renal failure (5/15), heart failure (3/15) and pulmonary edema (2/15), DIC (1/15), amniotic fluid embolism (1/15), metabolic encephalopathy (1/15), placental abruption (1/15) | 32.5 ± 3.23 | not reported |
| 6 | 20 - 33^+6^ (diagnosis) | n = 6  1: thalassemia (Bart's hydrops fetalis syndrome)  1: TTTs  1: Pavovirus B19  1: neck cystic lymphangioma  2: unknown causes | severe fetal hydrops, placental hydrops (3/6) | **Manifestations:** edema, anemia and hemodilution, hypoproteinemia, hypertension (3/6), temporary hyperthyroidism (3/6), proteinuria (2/6), elevated liver enzymes (2/6)  **Complications:** heart failure (2/6), pulmonary edema (1/6), renal insufficiency (1/6), respiratory failure (1/6) | 20^+1^ - 34^+2^ | 2: intrauterine death  2: induction of labor, died  2: died after delivery |
| 7 | 16 - 33 | n = 11  5: TTTs  1: heart malformation  1: sacrococcygeal teratoma  1: placental choriocarcinoma  1: gastrointestinal tumor  1: twin anemia-polycythemia sequence (TAPS)  1: selected intrauterine growth restriction (sIUGR) | hydrops fetalis, ascites , hydrothorax, hydropic placenta or placenta (1/11), polyhydramnios | **Manifestations:** edema, anemia and hemodilution, hypoproteinemia (10/11), hypertension (5/11), proteinuria (4/11), elevated liver enzymes (1/11), elevated urea nitrogen (1/11)  **Complications:** none | 23 - 36 (9/11) | 6: intrauterine death  1: died 10 min after delivery  2 (TTTs): cesarean section (at 34 and 36 weeks, respectively), survived  2 (TTTs): alive, not delivered until publication (33 and 35 weeks, respectively) |
